# 3D Printing with the Commercial UV-Curable Standard Blend Resin: Optimized Process Parameters towards the Fabrication of Tiny Functional Parts

**DOI:** 10.3390/polym11020292

**Published:** 2019-02-09

**Authors:** Valentina Bertana, Giorgio De Pasquale, Sergio Ferrero, Luciano Scaltrito, Felice Catania, Carmelo Nicosia, Simone L. Marasso, Matteo Cocuzza, Francesco Perrucci

**Affiliations:** 1 Chilab-Materials and Microsystems Laboratory, DISAT, Politecnico di Torino-Via Lungo Piazza d’Armi 6, 10034 Chivasso (Turin), Italy; sergio.ferrero@polito.it (S.F.); luciano.scaltrito@polito.it (L.S.); felice.catania@polito.it (F.C.); carmelo.nicosia@polito.it (C.N.); simone.marasso@polito.it (S.L.M.); matteo.cocuzza@infm.polito.it (M.C.); francesco.perrucci@polito.it (F.P.); 2 DIMEAS, Politecnico di Torino, Corso Duca degli Abruzzi 24, 10129 Turin, Italy; giorgio.depasquale@polito.it; 3 CNR-IMEM, Parco Area delle Scienze, 37a, 43124 Parma, Italy

**Keywords:** SL process, hatching effect, post-curing time, pigment, mechanical resistance, build accuracy

## Abstract

Stereolithography 3D printing is today recognized as an effective rapid prototyping technique in the field of polymeric materials, which represents both the strengths and the weaknesses of this technique. The strengths relate to their easy handling and the low energy required for processing, which allow for the production of structures down to the sub-micrometric scale. The weaknesses are a result of the relatively poor mechanical properties. Unfortunately, the choice of the right material is not sufficient, as the printing parameters also play a crucial role. For this reason, it is important to deepen and clarify the effect of different printing conditions on final product characteristics. In this paper, the behavior of commercial Standard Blend (ST Blend) acrylic resin printed with stereolithography (SL) apparatus is reported, investigating the influence of printing parameters on both the tensile properties of the printed parts and the build accuracy. Twenty-four samples were printed under different printing conditions, then dimensional analyses and tensile tests were performed. It was possible to find out the optimum printing setup to obtain the best result in terms of mechanical resistance and printing accuracy for this kind of resin. Finally, a micrometric spring was printed under the optimal conditions to demonstrate the possibility of printing accurate and tiny parts with the commercial and inexpensive STBlend resin.

## 1. Introduction

The term “additive manufacturing” refers to a layer-by-layer building method for prototypes 3D printing. Stereolithography (SL), selective laser sintering (SLS), and fused deposition modelling (FDM) are some examples of additive manufacturing technologies. 

Among the cited techniques, SL is surely the oldest one if we consider the pioneering work of Charles W. Hull in 1986, in which he outlined a system for building objects from a liquid medium [1]. Since then, many researchers have studied methods to improve this approach and to exploit this technology with broad-spectrum applications. Nowadays, SL is involved in several application fields, such as bioengineering (prostheses [2] and pre-surgical phantoms [3], interfaces for the human body [4]), microfluidics [5], labs-on-chip (LOC) [6,7], Micro Electro Mechanical System (MEMS) [8], chemical laboratory analysis, and electronic and mechanical devices (circuit-like structures [9,10], electronic components [11], electrochemical cells case [12], antennas [13,14], energy harvesters [15], dampers, connectors, etc.). The goal is to further enlarge the family of SL applications finding the optimal printing procedure for each case, which mainly relies on material composition and printing parameters. With this in mind, the commercial acrylic resin STBlend from FunToDo was characterized after SL printing and optimal printing parameters were found. The low-cost STBlend resin was chosen for some of its interesting properties, also reported in literature. Indeed, STBlend can be employed to prepare fiber-reinforced matrixes [16,17], and it was demonstrated to be a good scatterer for ultrasound applications [18] and inert for standard polymerase chain reaction (PCR) analyses [6]. Moreover, as reported in [6], parts printed with STBlend are suitable for high-temperature applications (up to 100 °C). This paper aims to add information about STBlend resin when polymerized with different printing parameters, pushing its performance in reproducing strong and microscopic parts (e.g., to be integrated in micro electro-mechanical systems, or MEMS) to the limit. Furthermore, a test examining how coloring pigment inside the resin affects object properties is reported. Indeed, it is known that, even in optimized commercial resins, the color is given by a pigment that might settle if the blend is not periodically mixed. Considering that pigment competes with photoinitiator during polymerization [19], it is likely it has an influence on dimensional accuracy and mechanical properties. Very few works have examined such issues in the field of stereolithography resins, limiting study to the investigation of curing characteristics and color [20] or final hardness properties [21]. Other works, like the one of Macarie and Ilia [19], focused mainly on acrylic coatings. In this work, by printing samples before and after pigment settling, more information is added about the influence of coloring pigment on dimensional and mechanical characteristics of the same stereolithography resin. 

Overall, this work aims not only to enrich the knowledge about a specific resin, but also adds data to the literature related to SL printing parameters. Indeed, it was observed that many researchers have worked on finding the optimal printing conditions for SL process in order to obtain high quality parts, both in terms of mechanical properties and dimensional accuracy (Table A1 summarizes the most relevant works in this field, highlighting the parameters considered and the material). But the influence of process parameters on dimensional accuracy and mechanical properties has never been considered together. Moreover, most of works have considered only epoxy-based resins. Epoxy-based resins are blends of epoxides with acrylate components. This mixture leads to the creation of interpenetrating networks during polymerization and is typical for commercial resins optimized for high printing accuracy. Indeed, epoxies are characterized by a slow cationic polymerization different from high reactive acrylates which polymerize following a fast, radical polymerization. Therefore, the presence of epoxides induces a lower stress due to shrinkage, resulting in more dimensionally accurate objects. However, epoxy-based resins are not always the right choice, from among the alternatives, due to their slow reaction and their exothermic polymerization process. These are some reasons why acrylate resins are sometimes preferred.

## 2. Materials and Methods

### 2.1. CAD Modeling

**Figure 1:**
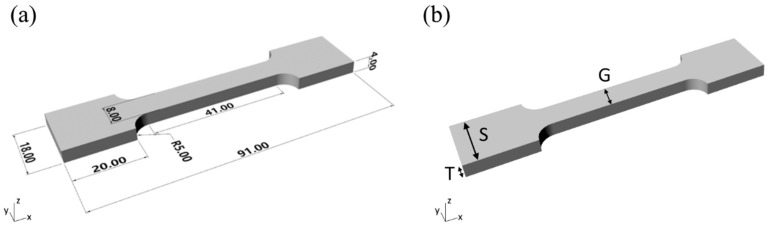
Tensile sample details. (**a**) Nominal dimensions (expressed in millimeters). (**b**) Measured thickness (T), gauge width (G), and shoulder width (S).

A tensile test sample in accordance with ISO527 [1,2,3,4,5] was designed to be used both for a dimensional check and, subsequently, a mechanical tensile test. It has a characteristic shape and it is usually called “dog-bone tensile piece”. As shown in Figure 1a, each sample is composed of two shoulders and one central part called “gauge” or “gage”. It is recommended the total length of the gage be at least four times the diameter (or the width) to avoid effects from the shoulders.

The sample geometry was generated in Solidworks^®^ and then converted in *.STL (Standard Triangulation Language) taking into account the dimensional constraints and the available printing area.

### 2.2. Printing Equipment and Material

In this work, customizable 3D printing equipment designed and fabricated by Microla Optoelectronics s.r.l. (Turin, Italy) was used. This printer enables the user to manually set the laser power and layer thickness (from 20 to 200 µm). Moreover, hatching direction, hatching spacing and hatching speed can be tuned. In particular, hatching speed can be set differently for each layer, as well as for its edges and its internal area. It has a 170 mm × 200 mm working area and exploits a 405 nm laser source to trigger the polymerization. The laser is mounted on a galvo-scanner and the final spot diameter focused on the building platform is 100 µm. Model slicing, setting of the parameters, and writing the instructions for G-code, were performed using the software BeamConstruct by HA Laser System (München, Germany).

The material used was the acrylic resin STBlend-Black from FunToDo (Alkmaar, The Netherlands), which is characterized by a 100 cP viscosity and 1016 g·dm^−3^ density.

### 2.3. Printing of the Samples

The SL process considered starts with a preparation step in which the vat is properly filled with liquid resin. After that, the object is built by polymerizing the resin layer-by-layer, thanks to the data provided by the slicing program. Twenty-four samples with different printing conditions were produced. Hatching direction (H-dir) and post-curing time (PCT) parameters, known to have an effect on the final mechanical properties, were varied in order to evaluate the influence on both dimensional accuracy and mechanical properties. As regards H-dir, along sample axis (x-dir) and orthogonal to sample axis (y-dir) were the two chosen levels. The selection of post-curing times was initially based on literature: indeed, 60 min PCT occurs frequently and was also found to be the best value for epoxy-based resins [22]. Then, a quicker PCT was chosen (14 min), aiming to shorten the whole printing process. Moreover, considering that the resin used was dark, thanks to a black pigment dispersed inside it, the effect of pigment, and thus resin transparency (Tr), was investigated by printing with a more opaque (Op) and pigmented resin (before pigment settling) and a clearer (Cl), less pigmented resin (after one month pigment settling, to ensure almost complete deposition). A design of experiment (DOE) with two levels for each parameter was introduced, as reported in Table 1. 

According to the DOE parameters, the eight combinations reported in Table 2 were attained for the fabrication of samples. For each combination, three samples were built and listed in the same table. Hence, twenty-four tensile pieces were obtained and identified by different numbers.

Other printing parameters, typical for SL process, were set constant. A layer thickness equal to 0.1 mm was chosen in accordance to Cho et al. [23] to reduce error on dimensional accuracy. Indeed, they found that once the hatch overcure parameter is fixed, by choosing the higher hatch spacing with the lower layer thickness, it is possible to obtain a low absolute error related to nominal dimensions. Therefore, they suggested a layer thickness equal to 0.1 mm and a hatch spacing higher than 0.22 mm. In this study, a lower hatch spacing (0.05 mm) was selected in order to obtain a tougher part. This choice was determined by the absence of printing supports, which caused adhesion of the sample first layer to the building platform and, therefore, a considerable effort to detach the object from the platform. The other fixed parameters, optimized for the resin in use, were: laser power density equal to 5.97 W·mm^−2^, scanning velocity (SV) for edges equal to 1000 mm·s^−1^, and SV for internal area equal to 2000 mm·s^−1^.

### 2.4. Post-Curing Treatment

Once the object printing was completed, the building platform was removed from the printer to detach the object and clean it with isopropyl alcohol (IPA), which allows the proper removal of uncured resin. Even if thermal post-curing produces the highest degree of curing [24] reducing heterogeneity and anisotropy [25], UV post-curing (with a 120 W nominal power UV lamp) was chosen because thermal post-curing induces undesired higher shrinkage strains [26] and represents an accelerated ageing mechanism [27]. Moreover, in order to overcome possible shrinkage during the static UV post-curing step, each specimen was exposed for half the post-curing time and then turned upside down to complete the curing.

### 2.5. Dimensional Characterization

Samples shoulder width (S), gauge width (G), and thickness (T) were measured as described in Figure 1b to evaluate dimensional errors. A micrometer gauge (Mitutoyo, range 0–25 mm, resolution 0.01 mm) was used. In detail, for each sample, the performed measurements were two for shoulder width and one for each shoulder. Three measurements were taken for gauge width: one in the middle of the gauge and the other two on its extremities. Five measurements were taken for thickness. Finally, average values for T, S, and G were calculated.

### 2.6. Mechanical Characterization

After dimensional characterization, the stress-strain curve was recorded for each of the twenty-four samples. Tests were performed at 0.5 mm/min velocity in controlled displacement mode at room temperature (25 °C) with a 10 kN load cell. Each tensile sample was properly clamped and tested using an MTS QTest 10 machine. Mechanical testing equipment acquires data at 10 Hz frequency and plots a real-time graph. Each point in the chart describes the load variation (expressed in Newtons) vs. the upper grip displacement (expressed in millimeters). Load and displacement data were processed to obtain a stress (σ)-strain (ε) curve for each specimen, according to Equations (1) and (2).

(1) $$\sigma =\frac{load}{area}$$

(2) $$\varepsilon =\frac{Displacement}{length}$$

According to nominal dimensions reported in Figure 1a, *area* is equal to 32 mm^2^ while *length* is equal to 51 mm. 

From the stress-strain curve, the characteristic Young’s modulus (E) was extrapolated.

### 2.7. Statistical Analysis of the Results

Analysis of variance (ANOVA) was performed to observe the influence of printing parameters on the final properties of the part. The influence of parameters on dimensional accuracy and Young’s modulus (E) was investigated since the aim was to find the optimal condition to build micrometric and mechanically strong parts. Thus, ANOVA was performed by observing the mean values (V_avg_) of E, T, S, and G, calculated as reported in Equation (3). Standard deviation was used to estimate the error (err), calculated as reported in Equation (4).

(3) $$v_{avg}=\sum_{k=1}^{n}\frac{v_{k}}{n}$$

(4) $$err=\sqrt{\sum_{k=1}^{n}\frac{{\left(v_{k}-v_{avg}\right)}^{2}}{n}}$$

First of all, results from dimensional and mechanical characterization were sorted to define twelve sample groups, as reported in Table 3. Each of the groups was made up of six samples characterized by two fixed printing parameters (according to the parameters combination reported in Table 2). Thus, the influence of the variable printing parameter could be evaluated by calculating the factor of influence (F) for E, T, S, and G measurements.

Considering each group separately, comprising two subgroups, F was calculated by dividing the variance among the group (v_among_^2^) with the variance inside each sub-group (v_inside_^2^). v_among_^2^ and v_inside_^2^ were calculated as reported in Equations (5) and (6).

(5) $$v_{among}^{2}=\frac{1}{sg-1}\sum_{k=1}^{sg}s\cdot {\left({\bar{V}}_{k}-\bar{V}\right)}^{2}$$

(6) $$v_{inside}^{2}=\frac{1}{s_{tot}-sg}\sum_{k=1}^{sg}D_{k}$$

where *sg* is the number of subgroups (always equal to 2 in this case), *s* is the number of samples for each subgroup (always equal to 3 in this case), ${\bar{V}}_{k}$ and $\bar{V}$ are respectively the mean value for subgroup k and the mean value of the group, *s_tot_* is the total number of samples in the group (always equal to 6 in this case), and *D_k_* is the deviance inside subgroup k.

Finally, the influence of each printing parameter was evaluated by comparing the F values calculated for its groups (e.g., for H-dir the F values calculated for groups a, b, c, and d) with critical value extrapolated from the F-distribution table for level of significance α = 0.05 taking into account the degrees of freedom associated to F, (1,4) in this case. Thus, for each printing parameter, if in more than 5% of cases F values are higher than the critical value (always equal to 7.71 in this study), it is said that the printing parameter has an influence on the printed part characteristic observed (E, T, S, or G in this case).

### 2.8. Micrometric Spring Printing

**Figure 2:**
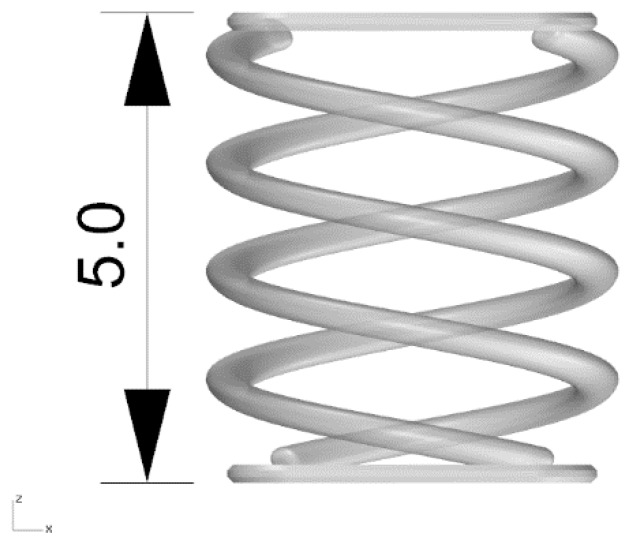
3D geometry of the micrometric spring. Dimension is expressed in mm.

The main goal of this work was to find a printing parameter setup to accurately build tiny parts. Thus, a small spring, which integrates a 500 µm diameter coil, was printed with the optimized printing parameters. The spring has an external diameter equal to 4.5 mm and is 5 mm high. The 3D geometry is reported in Figure 2.

## 3. Results

The inaccuracies intrinsically connected with SL processing are mainly due to various sources [28]: integration of CAD/CAM (Computer Aided Design/Computer Aided Manufacturing), materials behavior after polymerization, laser spot diameter, manufacturing parameters, and post-processing steps, associated also to accidental and systematic errors. 

Previous works demonstrated that dimensional accuracy in SL is worse along *z*-direction than on the *x*-*y* plane [29]. These inaccuracies are mainly due to shrinkage, a complex phenomenon that may interest the object both during printing and post-processing steps [23]. For the printing process considered in this study, shrinkage can firstly occur during polymerization in the resin vat causing the so-called “curl distortion” [30]. If the parameters are optimized and a sufficient curing degree is reached within the printing process, the shrinkage induced by post-curing treatment will be less relevant. Indeed, the higher the curing during printing, the lower the shrinkage during post-curing [31]; the latter has a large influence on mechanical resistance of final objects [22] leading to higher values of elastic modulus than green samples [24].

### 3.1. Dimensional Characterization

**Figure 3:**
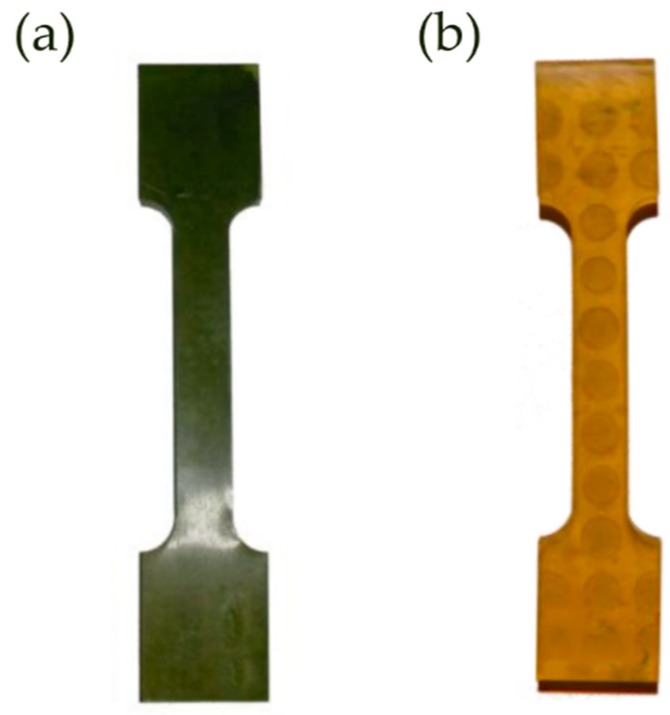
Two examples of dog-bone tensile samples. (**a**) Sample printed with Op resin. (**b**) Sample printed with Cl resin.

Two of the dog-bone tensile samples obtained after printing and post-curing are reported in Figure 3, one for Op resin (Figure 3a) and one for Cl one (Figure 3b).

**Figure 4:**
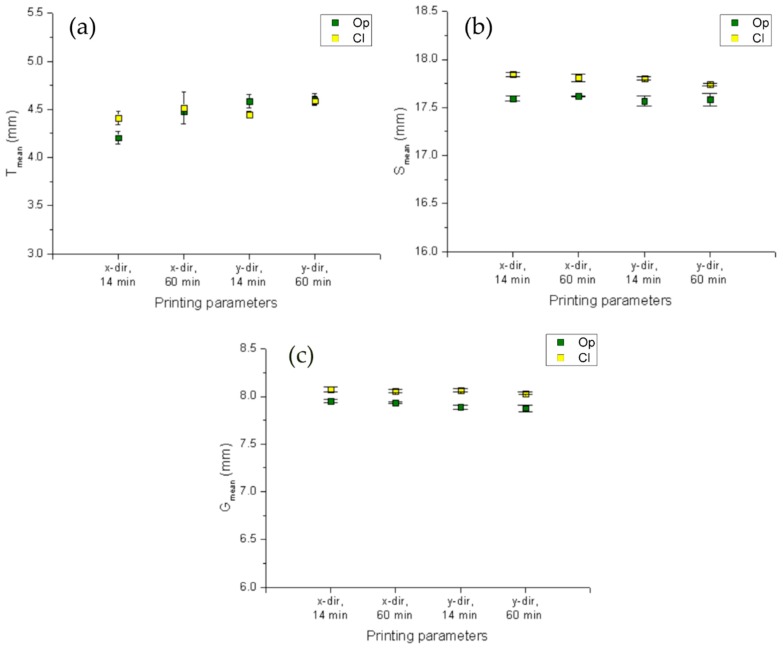
Plot of mean values of (**a**) T, (**b**) S, and (**c**) G both for Op and Cl resin. On the y axis, the same 2.5 units value span is reported to allow comparisons.

The plots collected in Figure 4, show the mean values for T (Figure 4a), S (Figure 4b), and G (Figure 4c) with corresponding error bars, and they are plotted by processing measurement data of the three samples related to each of the cases reported on horizontal axis. A comparison between Op and Cl resin is also performed and the results are collected in the same plots. 

One of the aims of the study was to observe the dimensional accuracy of the printed objects and its dependence on the printing parameters set. It seems that no evidence of hatching direction or post-curing time influence on T, S, or G could be reported. Instead, resin transparency affects dimensional accuracy inducing a wider polymerized strand with respect to darker resin, resulting in wider objects. This effect seems to compensate for the effect of shrinkage, obtaining sample widths closer to the nominal values. Finally, using the same value span along the y-axis of the three histograms in Figure, it is possible to prove that, also for the printing setup employed here, major errors occur along z-direction (thickness), as reported elsewhere [29]. Indeed, all samples (independently from the resin type and process parameters considered) show that T measurements have a higher standard deviation than S and G measures. 

### 3.2. Mechanical Characterization

**Figure 5:**
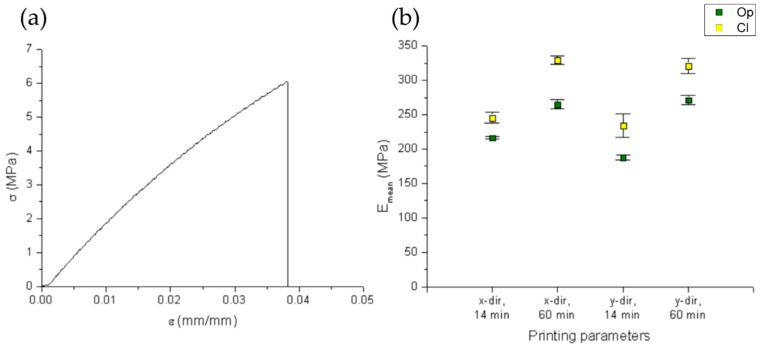
Results from mechanical characterization. (**a**) Typical stress-strain curve for the tested samples (here the one relative to sample #4). (**b**) Plot of mean values for Young’s modulus.

As for dimensional measurements, data were collected in plots showing the mean values of the Young’s modulus (Figure 5b). Figure 5a shows a typical stress-strain curve obtained from the tensile testing.

The mechanical characterization of material properties, according to the reported DOE, revealed that all samples (independently from the resin type and process parameters considered) showed a brittle behavior with low permanent deformation after sample break. For all tensile specimens, the hatching direction seems to have no influence on mechanical properties. A similar result has been obtained with epoxy-based resins and is in accordance with previous studies [32] which have demonstrated that object layout (if it is built flat or on edge) is more influential than object orientation on the horizontal plane, which has almost no influence. Instead, the post-curing time seemed to have an effect on mechanical behavior: for both clear and opaque resin, the higher the post-curing time, the higher the Young’s modulus. This is in line with the study of Chockalingam et al. [22] in which, for an epoxy resin, it was found that a 60 min PCT is the best choice for enhanced tensile strength of SL parts. In addition, resin transparency affects the mechanical properties, resulting in higher mean Young’s modulus values for clear resin, regardless of printing direction and post-curing time. 

### 3.3. Statistical Analysis of the Results

**Figure 6:**
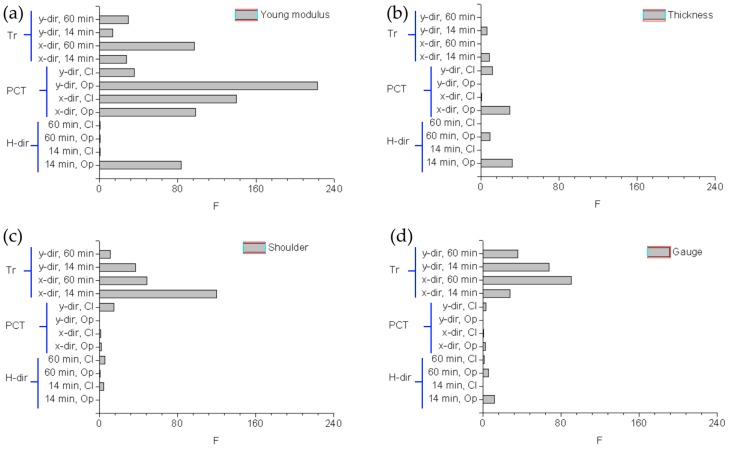
ANOVA: plot of the F values computed for (**a**) Young’s modulus, (**b**) thickness, (**c**) shoulder width, (**d**) gauge width.

The F values obtained for Young’s modulus (Figure 6a), thickness (Figure 6b), shoulder width (Figure 6c), and gauge widths (Figure 6d) are schematically reported in Figure 6. The comments previously reported are all proved: post-curing time and transparency have a significant impact on the final Young’s modulus of the STBlend printed objects (with respective F mean values equal to 124.37 and 42.13). As regards printing accuracy, polymer transparency is influential for planar dimensions, thus impacting on shoulder and gauge width (with respective F mean values equal to 54.38 and 55.71).

### 3.4. Micrometric Spring Printing

**Figure 7:**
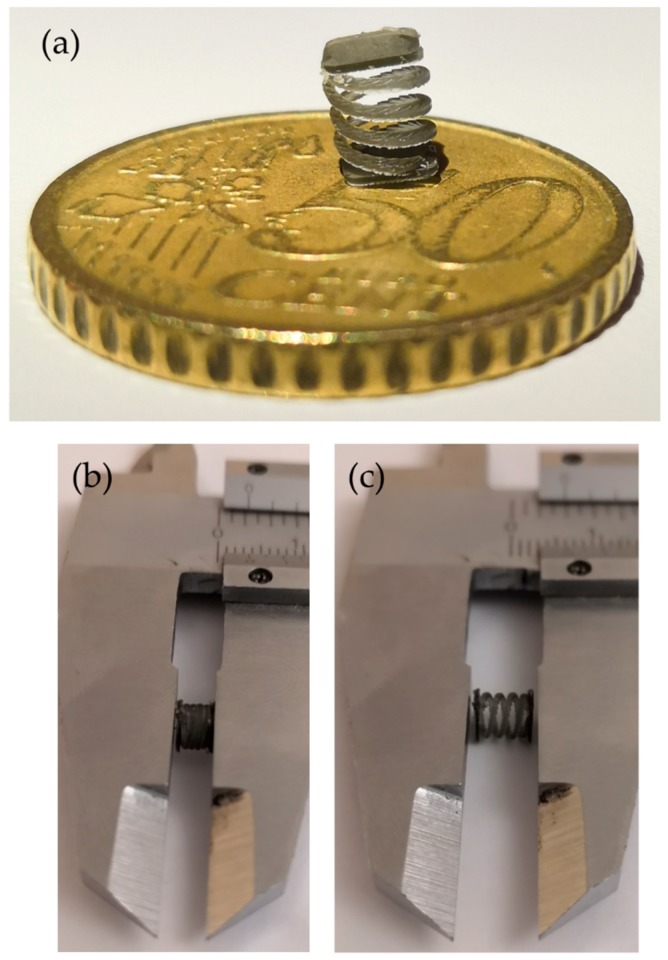
SL printed micrometric spring. (**a**) Geometry details, (**b**) compression, (**c**) release.

Following the previous results, the spring was printed with Op resin, which showed more repeatable results than Cl resin, setting y-dir and 60 min post-curing time to have the higher Young’s modulus. As visible is Figure 7a, the geometry appears almost identical to the CAD drawing; moreover, the spring can be compressed (Figure 7b) and is finally able to recover its shape (Figure 7c).

For this kind of application, a lower layer thickness would be suggested; indeed, from Figure 7a, the layers are clearly visible along the coil.

## 4. Discussion

At the end of this work, we can state that the first hypotheses deduced from the experimental results were verified by the analysis of variance. As regards the mechanical properties of the objects printed with STBlend resin, they were found to be mainly affected by post-curing time and resin transparency. During the printing process, the laser does not stimulate the complete polymerization of the resin but creates a sort of “scaffold” with the desired shape in order to speed-up the printing process. Then, a post-curing step is required in order to fully cure the un-polymerized blend trapped in the printed scaffold. Thus, the longer the post-curing time, the higher the curing degree and the polymer strength, until complete polymerization is reached. Therefore, surely the post-curing process induces a further hardening of the resin, both for clear and opaque resin. The higher values of Young’s modulus for clear resin could be due to both the small number of weakening resin/pigment interfaces but also to the better UV light penetration inside the object, which allows for a higher curing degree at higher depths inside the parts (if compared with the dark resin layers which can act as a shield for the inner volumes of the object). As regards printing accuracy, a possible explanation of the influence of resin transparency on the planar dimensions of the samples came from experimental observation. All the measured values of shoulder width and gauge width for clear resin were higher than for the opaque ones. Again, UV light penetration inside the resin seems to be the reasonable explanation. During SL printing process, a laser spot hits the surface of the resin and continuously polymerizes the blend along a pre-determined path (which at the end generates the layer geometry). The width of the path is strictly dependent on the laser spot and the transferred energy; it is known for the spot to have a gaussian energy distribution, with higher values around its center and lower values at the boundaries. Therefore, there is an energy threshold value that determines the activation of the photoinitiator dispersed inside the resin. In the case of opaque resin, the pigment, as previously stated, could act as a shield for UV light, meaning only the volume directly around the center of the laser spot will have a sufficient energy dose to polymerize the resin. Thus, the area polymerized in opaque resin will be tighter than the one polymerized with the same printing parameters in clear, and almost transparent, resin.

In conclusion, the pigment seems to allow for a more accurate and spatially controlled polymerization, thus resulting in more similar printed parts and more repeatable results.

## 5. Conclusions

In this study, the commercial resin STBlend in two different forms (Op and Cl, depending on pigment sedimentation stage) was analyzed.

Dimensional accuracy and mechanical behavior were evaluated tuning printing parameters. It was observed that dimensional accuracy is higher on the x-y plane than along z-direction. Moreover, in the presented printing setup, hatching direction and post-curing time parameters seem not to influence dimensional accuracy, which conversely is affected by resin transparency. As regards mechanical properties of printed samples, post-curing time and resin transparency have an impact on mechanical properties, while hatching direction has almost no effect.

Finally, the performances of the resin treated with the optimized parameters was assessed by printing a micrometric spring, which could be suitable for integration inside a millimetric mechanical actuation system.

## Figures and Tables

**Table 1:** Design of experiment (DOE): Considered printing parameters and respective values setting.

Parameter *	Level 1	Level 2
*PCT*	14 min (7 min per side)	60 min (30 min per side)
*Tr*	Op	Cl
*H-dir*	x-dir 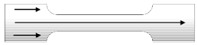	y-dir 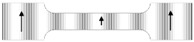

* Legend: PCT = post curing time; Tr = transparency; H-dir = hatching direction.

**Table 2:** Combination (Comb) of constructive settings for samples according to DOE.

Comb. #	H-dir	PCT	Tr	Samples #
1	x	14 min	Op	1 to 3
2	x	60 min	Op	4 to 6
3	y	14 min	Op	7 to 9
4	y	60 min	Op	10 to 12
5	x	14 min	Cl	13 to 15
6	x	60 min	Cl	16 to 18
7	y	14 min	Cl	19 to 21
8	y	60 min	Cl	22 to 24

**Table 3:** Sample groups for ANOVA.

Parameters Combination		Fixed Values							
		H-dir		PCT		Tr			
		x	y	14	60	Op	Cl	Group	Comb. #
Factor of influence (F)	**H-dir**	-	-	✓		✓		a	1-3
		-	-	✓			✓	b	5-7
		-	-		✓	✓		c	2-4
		-	-		✓		✓	d	6-8
	**PCT**	✓		-	-	✓		e	1-2
		✓		-	-		✓	f	5-6
			✓	-	-	✓		g	3-4
			✓	-	-		✓	h	7-8
	**Tr**	✓		✓		-	-	i	1-5
		✓			✓	-	-	l	2-6
			✓	✓		-	-	m	3-7
			✓		✓	-	-	n	4-8

## Appendix A

**Table A1:** Literature review on influence of printing parameters on mechanical properties and dimensional accuracy.

Literature			Investigated Parameters *							
Influence on:	Year	Author	LT	HS	SV	O	HO	LP	PC	Resin Used
*Mechanical properties*	1992	Jacobs [33]	x		x	x		x		NR*
	1997	Schaub et al. [34]	x			x	x			NR
	2004	Hague et al. [27]				x			x	Epoxy-based
	2006	Chockalingam et al. [35]	x			x			x	Epoxy-based
	2008	Chockalingam et al. [22]	x			x			x	Epoxy-based
	2009	Salmoria et al. [25]		x					x	Epoxy-based
	2014	Raju et al. [36]	x	x		x				Epoxy-based
	2014	Bangalore et al. [37]	x	x		x				Epoxy-based
*Dimensional accuracy*	1997	Rahmati et al. [38]	x	x			x			Epoxy-based
	1998	Onuh et al. [39]	x	x			x			Acrylic-based
	2000	Zhou et al. [28]	x	x			x			NR
	2000	Cho et al. [23]	x	x			x			Epoxy-based
	2001	Lee et al. [40]	x	x			x			NR
	2016	Chockalingam et al. [41]	x	x			x			Epoxy-based

* Legend: LT = layer thickness, HS = hatching spacing, SV = scanning velocity, O = orientation, HO = hatching overcure, LP = laser power, PC = post-curing, NR = not reported.

## References

[1] Hull C.W. (1986). Apparatus for Production of Three-Dimensional Objects by Stereolithography. U.S. Patent.

[2] De Pasquale G. (2016). Artificial human joint for the characterization of piezoelectric transducers in self-powered telemedicine applications. Meccanica.

[3] Vecchio M.D., Fea A.M., Spinetta R., Marasso S.L., Cocuzza M., Scaltrito L., Canavese G. (2017). Behaviour of the intraocular pressure during manual and vented gas forced infusion in a simulated pars plana vitrectomy. Int. J. Appl. Eng. Res..

[4] De Pasquale G., Kim S.-G., De Pasquale D. (2015). GoldFinger: Wireless human-machine interface with dedicated software and biomechanical energy harvesting system. IEEE/ASME Trans. Mechatron..

[5] Vaezi M., Seitz H., Yang S. (2013). A review on 3D micro-additive manufacturing technologies. Int. J. Adv. Manuf. Technol..

[6] Bertana V., Potrich C., Pirri C.F., Pederzolli C. (2018). 3D-printed microfluidics on thin poly(methyl methacrylate) substrates for genetic applications. J. Vac. Sci. Technol. B Nanotechnol. Microelectron. Mater. Process. Meas. Phenom..

[7] Perrucci F., Bertana V., Marasso S.L., Scordo G., Ferrero S., Pirri C.F., Cocuzza M., El-Tamer A., Hinze U., Chichkov B.N. (2018). Optimization of a suspended two photon polymerized microfluidic filtration system. Microelectron. Eng..

[8] Mao M., He J., Li X., Zhang B., Lei Q., Liu Y., Li D. (2017). The emerging frontiers and applications of high-resolution 3D printing. Micromachines.

[9] Gonzalez G., Chiappone A., Roppolo I., Fantino E., Bertana V., Perrucci F., Scaltrito L., Pirri F., Sangermano M. (2017). Development of 3D printable formulations containing CNT with enhanced electrical properties. Polymer.

[10] Scordo G., Bertana V., Scaltrito L., Ferrero S., Cocuzza M., Marasso S.L., Romano S., Sesana R., Catania F., Pirri C.F. (2019). A novel highly electrically conductive composite resin for stereolithography. Mater. Today Commun..

[11] Yang Y., Chen Z., Song X., Zhu B., Hsiai T., Wu P., Xiong R., Shi J., Chen Y., Zhou Q. (2016). Three dimensional printing of high dielectric capacitor using projection based stereolithography method. Nano Energy.

[12] Tanwilaisiri A., Zhang R., Xu Y., Harrison D., Fyson J. A manufacturing process for an energy storage device using 3D printing. Proceedings of the 2016 IEEE International Conference on Industrial Technology (ICIT).

[13] Nassar I.T., Weller T.M. An electrically-small, 3-D cube antenna fabricated with additive manufacturing. Proceedings of the 2013 IEEE Topical Conference on Wireless Sensors and Sensor Networks (WiSNet).

[14] Massaccesi A., Pirinoli P., Bertana V., Scordo G., Marasso S.L., Cocuzza M., Dassano G. (2018). 3D-Printable Dielectric Transmitarray With Enhanced Bandwidth at Millimeter-Waves. IEEE Access..

[15] De Pasquale G., Uttamchandani D. (2013). Energy harvesters for powering wireless systems. Handbook of MEMS for Wireless and Mobile Applications.

[16] Hofstätter T., Pedersen D.B., Tosello G., Hansen H.N. (2017). Applications of Fiber-Reinforced Polymers in Additive Manufacturing. Procedia CIRP.

[17] Hofstaetter T., Pedersen D.B., Nielsen J.S., Mischkot M., Hansen H.N. Investigation of digital light processing using fibre-reinforced polymers. Proceedings of the Euspen’s 16th International Conference & Exhibition.

[18] Füzesi K., Gyöngy M. (2017). Comparison of Two Inexpensive Rapid Prototyping Methods for Manufacturing Filament Target Ultrasound Phantoms. Ultrasound Med. Biol..

[19] Macarie L., Ilia G. (2007). Influence of pigment properties on UV-curing efficiency. J. Appl. Polym. Sci..

[20] Im Y.G., Chung S.I., Son J.H., Jung Y.D., Jo J.G., Jeong H.D. (2002). Functional prototype development: Inner visible multi-color prototype fabrication process using stereo lithography. J. Mater. Process. Technol..

[21] Gong H.-J., Mao-Lu W., Yang W. (2013). Surface Hardness Evaluation of Components Made by Color Stereolithography. J. Shanghai Jiaotong Univ..

[22] Chockalingam K., Jawahar N., Chandrasekar U., Ramanathan K.N. (2008). Establishment of process model for part strength in stereolithography. J. Mater. Process. Technol..

[23] Cho H.S., Park W.S., Choi B.W., Leu M.C. (2000). Determining optimal parameters for stereolithography processes via genetic algorithm. J. Manuf. Syst..

[24] Salmoria G.V., Ahrens C.H., Fredel M., Soldi V., Pires A.T.N. (2005). Stereolithography somos 7110 resin: Mechanical behavior and fractography of parts post-cured by different methods. Polym. Test..

[25] Salmoria G.V., Ahrens C.H., Beal V.E., Pires A.T.N., Soldi V. (2009). Evaluation of post-curing and laser manufacturing parameters on the properties of SOMOS 7110 photosensitive resin used in stereolithography. Mater. Des..

[26] Karalekas D., Aggelopoulos A. (2003). Study of shrinkage strains in a stereolithography cured acrylic photopolymer resin. J. Mater. Process. Technol..

[27] Hague R., Mansour S., Saleh N., Harris R. (2004). Materials analysis of stereolithography resins for use in Rapid Manufacturing. J. Mater. Sci..

[28] Zhou J.G., Herscovici D., Chen C.C. (2000). Parametric process optimization to improve the accuracy of rapid prototyped stereolithography parts. Int. J. Mach. Tools Manuf..

[29] Lynn-Charney C., Rosen D.W. (2000). Usage of accuracy models in stereolithography process planning. Rapid Prototyp. J..

[30] Horton L., Gargiulo E., Keefe M. (1993). An experimental study of the parameters affecting curl in parts created using stereolithography. Solid Freeform Fabrication Symposium.

[31] Wang W.L., Cheah C.M., Fuh J.Y.H., Lu L. (1996). Influence of process parameters on stereolithography part shrinkage. Mater. Des..

[32] Quintana R., Choi J.W., Puebla K., Wicker R. (2010). Effects of build orientation on tensile strength for stereolithography- manufactured ASTM D-638 type i specimens. Int. J. Adv. Manuf. Technol..

[33] Jacobs P.F. (1992). Rapid Prototyping & Manufacturing: Fundamentals of Stereolithography.

[34] Schaub D.A., Chu K.-R., Montgomery D.C. (1997). Optimizing stereolithography throughput. J. Manuf. Syst..

[35] Chockalingam K., Jawahar N., Ramanathan K.N., Banerjee P.S. (2006). Optimization of stereolithography process parameters for part strength using design of experiments. Int. J. Adv. Manuf. Technol..

[36] Raju B.S., Shekar U.C., Venkateswarlu K., Drakashayani D.N. (2014). Establishment of Process Model for Rapid Prototyping Technique (Stereolithography) to Enhance the Part Quality by Taguchi Method. Procedia Technol..

[37] Bangalore R., Gowda S., Udayagiri C.S., Narendra D.D. (2014). Studies on the Process Parameters of Rapid Prototyping Technique (Stereolithography) for the Betterment of Part Quality. Int. J. Manuf. Eng..

[38] Rahmati S., Dickens P. (1997). Stereolithography for injection mould tooling. Rapid Prototyp. J..

[39] Onuh S.O., Hon K.K.B. (1998). Application of the Taguchi method and new hatch styles for quality improvement in stereolithography. Proc. Inst. Mech. Eng. Part B J. Eng. Manuf..

[40] Lee S.H., Park W.S., Cho H.S., Zhang W., Leu M.C. (2001). A neural network approach to the modelling and analysis of stereolithography processes. Proc. Inst. Mech. Eng. Part B J. Eng. Manuf..

[41] Chockalingam K., Jawahar N., Chandrasekhar U., Praveen J., Karthic M. (2016). Development of Process Model for Optimal Selection of Process Parameters for Geometric Tolerances and Surface Roughness in Stereolithography. Int. J. Adv. Des. Manuf. Technol..

